# Regulation of Intestinal Epithelial Calcium Transport Proteins by Stanniocalcin-1 in Caco2 Cells

**DOI:** 10.3390/ijms17071095

**Published:** 2016-07-09

**Authors:** Jinmei Xiang, Rui Guo, Chunyun Wan, Liming Wu, Shijin Yang, Dingzong Guo

**Affiliations:** 1 College of Veterinary Medicine, Huazhong Agricultural University, Wuhan 430070, Hubei, China; hbswkjxjm@163.com (J.X.); wanchunyun16@163.com (C.W.); hnzdwlm@163.com (L.W.); hznydxysj@163.com (S.Y.); 2 Department of Animal Science, Hubei Vocational College Of Bio-Technology, Wuhan 430070, Hubei, China; 3 Hubei Key Laboratory of Embryo and Molecular Breeding, Hubei Academy of Agricultural Sciences, Wuhan 430064, Hubei, China; hbnkyguorui@163.com; 4 College of Animal Science, Yangtze University, Jingzhou 434023, Hubei, China

**Keywords:** calcium, intestinal epithelium, stanniocalcin-1, *TRPV5*, *TRPV6*

## Abstract

Stanniocalcin-1 (STC1) is a calcium and phosphate regulatory hormone. However, the exact molecular mechanisms underlying how STC1 affects Ca^2+^ uptake remain unclear. Here, the expression levels of the calcium transport proteins involved in transcellular transport in Caco2 cells were examined following over-expression or inhibition of STC1. These proteins include the transient receptor potential vanilloid members (*TRPV*) 5 and 6, the plasma membrane calcium ATPase 1b (*PMCA1b*), the sodium/calcium exchanger (*NCX1*), and the vitamin D receptor (*VDR*). Both gene and protein expressions of *TRPV5* and *TRPV6* were attenuated in response to over-expression of STC1, and the opposite trend was observed in cells treated with siRNA_STC1_. To further investigate the ability of STC1 to influence *TRPV6* expression, cells were treated with 100 ng/mL of recombinant human STC1 (rhSTC1) for 4 h following pre-transfection with siRNA_STC1_ for 48 h. Intriguingly, the increase in the expression of *TRPV6* resulting from siRNA_STC1_ was reversed by rhSTC1. No significant effect of STC1 on the expression of *PMCA1b*, *NCX1* or *VDR* was observed in this study. In conclusion, the effect of STC1 on calcium transport in intestinal epithelia is due to, at least in part, its negative regulation of the epithelial channels *TRPV5/6* that mediate calcium influx.

## 1. Introduction

Calcium (Ca^2+^) is an essential ion required for critical physiological processes in almost all organisms. Therefore, maintaining Ca^2+^ homeostasis is of vital importance. In mammals, Ca^2+^ is absorbed across intestinal or renal epithelia by two routes: a passive, non-saturable, poorly regulated paracellular process, and an active, saturable, highly regulated transcellular process [1]. The transcellular route requires energy and occurs predominantly in the proximal small intestine, renal distal convoluted tubules and the connecting tubules in response to Ca^2+^ demands [2]. In contrast, paracellular transport takes place throughout the length of the intestine and renal tubules, being responsible for the bulk of Ca^2+^ (re-)absorption in a concentration-dependent diffusion manner that does not consume energy [2].

Transcellular Ca^2+^ transport across intestinal or renal epithelia involves three steps. First, Ca^2+^ influx occurs at the apical membrane via epithelial Ca^2+^ channels (ECaC), which include the transient receptor potential vanilloid (*TRPV*) members 5 and 6 [1]. This step is considered to be the rate-limiting step for transcellular Ca^2+^ transport [1]; Second, intracellular diffusion is facilitated by vitamin D-dependent Ca^2+^-binding proteins calbindin-D_9K_ and calbindin-D_28K_ [3]; Last, extrusion at the basolateral membrane is achieved by either the Na^+^/Ca^2+^ exchanger *NCX1* or the plasma membrane calcium ATPase (*PMCA*) 1b [4]. The entire process is regulated by 1,25-dihydroxyvitamin D [1,25(OH)_2_D_3_], whose activity is mediated by a vitamin D receptor (*VDR*) [5].

In mammals, *TRPV6* is expressed in small intestine, kidney and exocrine tissues, while *TRPV5* is predominantly expressed in kidney and human syncytiotrophoblasts [6]. Calbindin-D_9K_ is present primarily in small intestines and kidneys (only mice), while calbindin-D_28K_ is present in kidneys, bones and brain [1]. *PMCA1b* is the predominant isoform of *PMCA* and is abundantly expressed in small intestines and other tissues [7], while *NCX1* is abundantly expressed in kidneys and at a low level in intestines [1]. These data indirectly suggest that transcellular Ca^2+^ transport across intestinal epithelia is predominantly mediated by *TRPV6*, calbindin-D_9K_ and *PMCA1b*, whereas *TRPV5*, calbindin-D_28K_ and *NCX1* are the principal components underlying renal Ca^2+^ re-absorption in mammals.

In mammals, extra- and intracellular Ca^2+^ concentrations are modulated by a complex homeostatic system including the hormones 1,25(OH)_2_D_3_, parathyroid hormone (PTH), and calcitonin [8]. However, stanniocalcin (STC) is considered as the main Ca^2+^/inorganic phosphate (Pi)-regulating hormone in fish, preventing gill and intestinal Ca^2+^ transport and promoting renal Pi re-absorption [9]. STC1, the mammalian homolog of fish STC, is expressed in multiple tissues and organs of many species and is involved in a variety of biological and pathological processes [10,11]. In contrast to its fish counterpart, STC1 is not detected in the circulatory system under normal circumstances except during gestation and lactation [12]. However, the regulatory effects of STC1 on Ca^2+^/Pi homeostasis are conserved from fish to mammals [13]. The exact molecular mechanism underlying how STC1 affects Ca^2+^ uptake has not been fully characterized. The purpose of this study was therefore to observe the effects of STC1 on the proteins mediating Ca^2+^ entry and extrusion in the intestines, and elucidate the mechanism of STC1-induced inhibition of Ca^2+^-absorption.

## 2. Results

### 2.1. Expression of STC1 in Transfected Caco2 Cells

**Figure 1:**
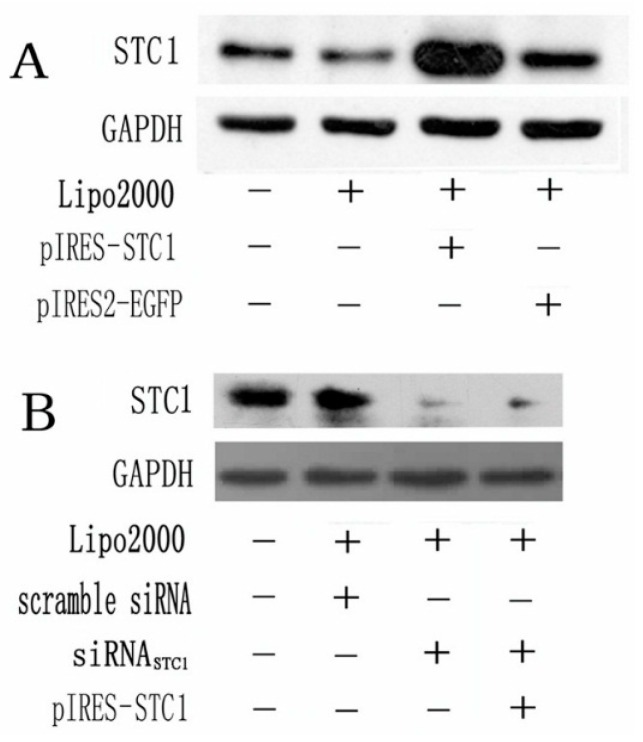
Detection of the expression of STC1 in Caco2 cells by Western blotting. (**A**) STC1 expression in Caco2 cells was detected 48 h post transfection of pIRES-STC1; (**B**) STC1 expression was detected 48 h post transfection of siRNA_STC1_, or pIRES-STC1 + siRNA_STC1_. All of the experiments were replicated for three times.

**Figure 2:**
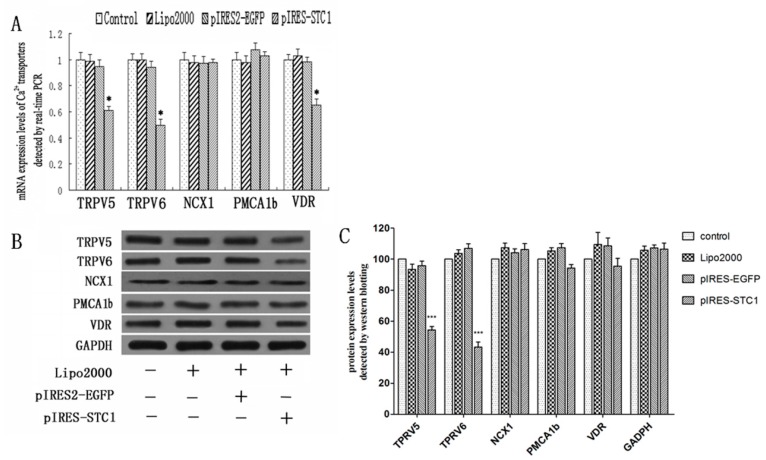
Analysis of transcellular calcium transport gene and protein expression levels in Caco2 cells transfected with pIRES-STC1. (**A**) Quantitative RT-PCR analysis of transcellular calcium transport genes (*n* = 4). Over-expression of STC1 reduced gene expression of *TRPV5*, *TRPV6* and *VDR*, with no effects on the expression of *NCX1* and *PMCA1b* genes (* *p* < 0.05 compared with control); (**B**) Western blotting analysis of transcellular calcium transport proteins. *TRPV5* and *TRPV6* protein expression levels were down-regulated by the over-expression of STC1. *NCX1*, *PMCA1b* and *VDR* proteins levels were not affected. All the experiments were replicated for three times; (**C**) Densitometric quantification of the Western blotting shown in (**B**). Each bar represents the means ± SD. (*n* = 3). *** *p* < 0.001 compared with control.

STC1 protein levels were detected by Western blotting. We found that the pIRES-STC1 vector was an effective vehicle for over-expressing STC1 protein and the expression was maintained at a high level after 48 h (Figure 1A). However, when cells were transfected with siRNA_STC1_ alone or with pIRES-STC1, STC1 protein expression was markedly blocked (Figure 2B). 

### 2.2. Effect of STC1 on the Expression of Calcium-Transporting Proteins

To determine the influence of STC1 over-expression on the regulation of Ca^2+^-transport proteins in Caco2 cells, cells were transfected with recombinant plasmid pIRES-STC1 for 48 h and subsequently analyzed. A marked decrease in the gene expression of *TRPV5*, *TRPV6* and *VDR* was identified by real-time RT-PCR in cells transfected with pIRES-STC1 when compared with control cells (*p* < 0.05; Figure 2A). No significant changes in the gene expression of *MCA1b* or *NCX1* were detected. Similarly, an obvious decrease of *TRPV5* and *TRPV6* expression was observed in pIRES-STC1 transfected cells (Figure 2B,C).

**Figure 3:**
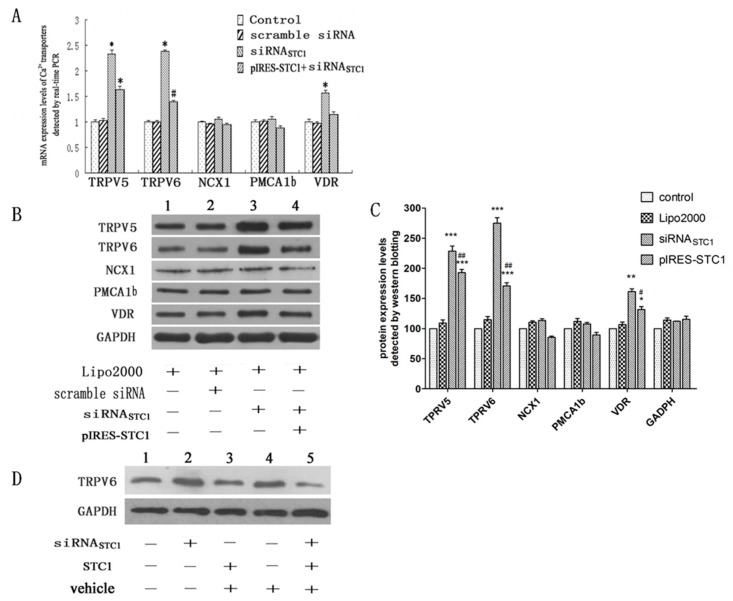
Analysis of epithelial Ca^2+^-transport protein expression in Caco2 cells after various treatments. (**A**,**B**) display quantitative PCR and western blotting analysis of the transport genes and proteins (*n* = 4). Both gene expression and protein levels of *TRPV5*, *TRPV6* and *VDR* were enhanced following 48 h transfection with siRNA_STC1_, and also with 48 h co-transfection with siRNA_STC1_ and pIRES-STC1 (with the exception of *VDR*). Expression of *NCX1* and *PMCA1b* revealed no significant change with inhibited expression of STC1 (* *p* < 0.01, ^#^ *p* < 0.05); (**C**) Densitometric quantification of the Western blotting shown in (**B**). Each bar represents the means ± SD. (*n* = 3). *** *p* < 0.001, ** *p* < 0.01, * *p* < 0.05 compared with control; ^##^ *p* < 0.01, ^#^ *p* < 0.05 compared with siRNA_STC1_ alone; (**D**) Western blotting analysis of *TRPV6* protein expression. *TRPV6* protein levels increased following transfection with siRNA_STC1_, and slightly decreased after a 4 h treatment with 100 ng/mL rhSTC1. However, treatment with rhSTC1 following siRNA_STC1_ transfection down-regulated the expression of *TRPV6*. All the experiments were replicated for three times.

To further characterize the response of Ca^2+^-transport proteins to STC1 in Caco2 cells, we designed an siRNA sequence (siRNA_STC1_) to inhibit the STC1 synthesis. Figure 3A revealed that *TRPV5*, *TRPV6* and *VDR* genes expression all significantly increased (*p* < 0.01) 48 h following siRNA_STC1_ transfection. Additionally, co-transfection of siRNA_STC1_ and pIRES-STC1 for 48 h also resulted in significant up-regulation of *TRPV5* and *TRPV6* genes expression (*p* < 0.01) (Figure 3A). Similar differences were not observed for *PMCA1b*, *NCX1* and *VDR* genes expression following co-transfection of pIRES-STC1 and siRNA_STC1_. Western blotting revealed a significant increase in the protein levels of *TRPV5*, *TRPV6* and *VDR* in response to siRNA_STC1_ treatment alone (Figure 3B,C), when co-transfected with pIRES-STC1, the levels of *TRPV5*, *TRPV6* and *VDR* were significantly decreased compared with siRNA_STC1_ treatment alone (Figure 3B,C). However, these treatments had no effect on *PMCA1b* and *NCX1* protein expression.

To complement the above findings, regulation of *TRPV6* expression by STC1 was investigated in further detail. Pre-treating cells with siRNA_STC1_ enhanced *TRPV6* protein expression, while exposure to 100 ng/mL of rhSTC1 for 4 h did not down-regulate *TRPV6* protein expression (Figure 3D). Intriguingly, cells exposed to 100 ng/mL rhSTC1 for 4 h following pre-treatment with siRNA_STC1_ for 48 h exhibited a marked attenuation of *TRPV6* protein expression when compared with siRNA_STC1_ treatment.

## 3. Discussion

The functions of STC1 have been extensively studied for decades, but many of them remain to be elucidated [14]. Unlike in fish, where STC functions as an anti-hypercalcemic hormone in a classical endocrine fashion [15], mammalian STC1 is normally undetectable in the blood and ubiquitously distributed in several tissues [16]. This implies that STC1 acts primarily as a local mediator of cell function in a paracrine/autocrine fashion. The Ca^2+^/Pi regulatory function of STC1 appears to have been maintained in mammals, although it has been described as a multi-functional hormone that is unlikely to play important roles in systemic Ca^2+^/Pi homeostasis [11,13,17,18]. However, there is limited information available concerning the relationship between STC1 and novel epithelial Ca^2+^ channels or transporters in mammals. Therefore, this study aimed to investigate the precise effects of STC1 on these proteins.

The digestive organs are important locations where STC1 influences Ca^2+^/Pi transport. Madsen et al. identified STC1 as a novel regulatory protein that decreased Ca^2+^ absorption and stimulated Pi absorption in swine and rat duodenum [17], providing direct evidence for the role of mammalian STC1 in the intestine in vitro. Here, we have examined the effects of STC1 over-expression and silencing on the regulation of proteins involved with Ca^2+^ entry and extrusion. Caco2 cells were employed as they have been widely employed to study duodenal transport processes given their resemblance to adult differentiated intestinal cells [19]. We have identified that over-expression of STC1 inhibits gene and protein expression of the epithelial Ca^2+^ channels *TRPV5/6*, particularly *TRPV6*, a principal mechanism of Ca^2+^ transport across intestinal epithelia. Furthermore, this inhibition could be removed by blocking STC1 expression via siRNA. Supporting this, exogenous rhSTC1 treatment abolished siRNA_STC1_-induced enhancement of *TRPV*6 expression. These findings support an inhibitory role for STC1 on *TRPV6* and *5* gene and/or protein expression, consistent with previous observations in zebrafish where *z*ECaC mRNA expression was enhanced following down-regulation of STC1 by morpholino microinjection [20], and in the human heart where STC1 functions as an L-channel blocker by mimicking the effect of nimodipine [21]. These data further support the hypothesis that ECaC form the primary hormone-regulated sites for active Ca^2+^ transepithelial transport in the intestine and renal tubules [1].

Consistent with previous findings obtained from work in fish [2,22], we did not observe changes to the levels of the transporters (*PMCA1b* and *NCX1*) that mediate Ca^2+^ efflux at the basolateral membrane, with changes to STC1 expression. Therefore, we speculate that STC1 does not affect ATP-dependent Ca^2+^-efflux, at least in intestinal cells. Additionally, given that it has been well established that Na^+^/Ca^2+^ exchange is not the major mechanism underlying Ca^2+^ efflux in enterocytes [23], the low impact of STC1 on *NCX1* expression in Caco2 cells is not surprising. Furthermore, a previous report has shown that STC1 can be up-regulated by 1,25(OH)_2_D_3_ in opossum kidneys [24]. However, we found that STC1 over-expression resulted in a significant inhibition of *VDR* mRNA levels as detected by real-time RT-PCR, while further marked effects on the protein expression were not investigated by the methods we employed. These data suggest that STC1 does not affect the functions of 1,25(OH)_2_D_3_ in Ca^2+^ transport across the absorptive epithelia; however, whether STC1 alters the concentration of 1,25(OH)_2_D_3_ in local cells and its synthesis in other sites remains unclear.

## 4. Materials and Methods

### 4.1. Cell Culture

Caco2 cells (ATCC HTB-37) were maintained in a high-glucose formulation of Iscove’s Modified Dulbecco’s Medium (IMDM, Hyclone, Logan, UT, USA) supplemented with 10% fetal bovine serum (Hyclone), 50 mg/mL penicillin G (Sigma, St. Louis, MO, USA), 50 mg/mL streptomycin sulfate (Sigma), and 4 mM glutamine (Sigma) and incubated at 37 °C in a humidified atmosphere containing 5% CO_2_. For experiments, cells were detached by 0.25% trypsin (Sigma) with 0.02% Ethylene Diamine Tetraacetic Acid (EDTA), seeded in six-well dishes (Nest Biotech, Shanghai, China) at a density of 2 × 10^5^ cells/well, and fed every other day with growth medium for 1–2 weeks to achieve a fully differentiated and attached cell phenotype prior to experimental treatments.

### 4.2. Construction of STC1 Expression Vector

Total RNA from Caco2 was isolated using TRIzol reagent (Invitrogen, Carlsbad, CA, USA) according to the manufacturer’s protocol. Total RNA samples (with A_260_/A_280_ ratios between 1.8 and 2.0) were quantified spectrophotometrically by absorbance at 260 nm. Less than 2 μg total RNA was used to generate cDNA using a reverse transcription kit (TaKaRa, Dalian, China).

The complete CDS of human STC1 mRNA (NM_003155.2) was amplified using the following primers: sense, 5′-ATC AAG CTT ATG CTC CAA AAC TCA G-3′; antisense, 5′-ATG GAT CCT TAT GCA CTC TCA TGG-3′ (*Hin*dIII *and Bam*H I cleavage sites are underlined). Amplicons were subjected to 1% agarose gel electrophoresis, purified using a DNA gel extraction kit (Axygen, Union City, CA, USA), and cloned into a pMD-18T TA-clone vector (TaKaRa) for sequencing and subsequent digestion by restriction endonucleases. The gene fragments of interest were then subcloned into the pIRES2-EGFP vector (Invitrogen) using T4 DNA ligase (TaKaRa). DNA sequencing was performed with an ABI Prism 310 genetic analyzer (Applied Biosystems, Foster, CA, USA). The recombinant plasmid was named pIRES-STC1. Endotoxin-free plasmid DNA was prepared from overnight cultures of *E. coli* DH5α (Invitrogen) containing pIRES-STC1 using the EZNA plasmid max kit (Omega, Doraville, GA, USA).

### 4.3. Design and Synthesis of siRNA

Small interfering RNA oligonucleotide duplex targeting human STC1 (siRNA_STC_) was designed and synthesized by Ribobio Biotech Co., Ltd. (Guangzhou, China) as follows: 5′-AUU CGG AGG UGC UCC ACU UdT dT-3′ (sense) and 5′-AAG UGG AGC ACC UCC GAA UdT dT-3′ (antisense). A functional non-coding siRNA (scrambled siRNA) and a siRNA targeting β-actin (siRNA_actin_, data not shown) were used as controls. The sequences of scrambled siRNA and siRNA_actin_ are proprietary knowledge of Ribobio Biotech.

### 4.4. Transfection and Treatments of Caco2 Cells

Caco2 cells were seeded in six-well plates (2 × 10^5^ cells/well) and incubated overnight in complete IMDM medium without antibiotics prior to transfection. Cells were transfected with pIRES-STC1 (4.0 μg/well) or equivalent amount of pIRES2-EGFP, and siRNA_STC1_ (100 pmol/well) or equivalent amount of scrambled siRNA, and the best volume ratio of pIRES-STC1 (1.0 μg/well) and siRNA_STC1_ (100 pmol/well) co-transfected into Caco2 cells using Lipofectamine 2000 (Invitrogen) according to the manufacturer’s protocol. Total RNA and protein were then harvested after 48 h.

To further investigate the role of STC1, Caco2 cells were first transfected with siRNA_STC1_ for 48 h followed by administration of rhSTC1 protein (100 ng/mL dissolved in Hanks’ balanced salt solution, ProSpec, Rehovot, Israel, HOR-259) for 4 h. Total RNA and total protein were then harvested. The dose of rhSTC1was chosen based on published report by Madsen et al., who demonstrated that the minimum intestinal net Ca^2+^ absorption and maximum conductance occurred with the addition of 100 ng/mL rhSTC1 [17]. 

### 4.5. Real-Time PCR 

Total RNA extraction and cDNA library preparation were performed for each sample as described above. Gene expression was measured by real-time PCR using an ABI StepOne™ Real-Time PCR System (Applied Biosystems). Reactions consisted of 1 × SYBR Green I (TaKaRa), 0.2 µM forward, reverse primers (Sangon, Shanghai, China) for each gene (Table 1), and 1/50 of total reaction volume of ROX. *GAPDH* was used as an internal control. After denaturation at 94 °C for 5 min, the reaction proceeded for 40 cycles of 94 °C for 15 s, 60 °C for 15 s and 72 °C for 25 s. A final 5-min extension step at 72 °C was performed.

### 4.6. Western Blotting

Cells were lysed for 10 min in ice-cold lysis buffer (50 mM Tris-HCl (pH 7.4), 150 mM NaCl, 1% Triton X-100, 1% SDS, 2 mM EDTA, 3% Nonidet P-40, 2 mM orthovanadate, 50 mM NaF, 10 mM NaPPi, and 10 mg/mL each of aprotinin and leupeptin) supplemented with 1 mM PMSF (Beyotime, Wuxi, China). The samples were centrifuged at 14,000× *g* for 15 min, and supernatants were collected. Total protein concentration was measured with a BCA Kit (Beyotime) following the manufacturer’s protocol. A total of 30 μg of protein from each sample was subjected to electrophoresis on 12% SDS/PAGE and transferred to a 0.22-μm PVDF membrane (Millipore, Bedford, MA, USA) that was pre-treated with methanol for 2 h before transfer. The membranes were blocked overnight in 5% (*w*/*v*) skim milk in TBST (0.01 M Tris-HCl (pH 7.5), 0.15 M NaCl, 0.5% Tween-20). Immunoblotting was conducted using diluted rabbit polyclonal antibodies against STC1 (1:1000, Abcam, Cambridge, MA, USA), *TRPV6* (1:200, ABclonal, Woburn, MA, USA), *TRPV5* (1:600, ABclonal), *NCX1* (SLC8A1, 1:400, ABclonal) and *VDR* (1:1000, ABclonal), mouse monoclonal antibodies against *PMCA1b* (ATP2B1, 1:300, Abgent, San Diego, CA, USA), and *GAPDH* (1:5000, Cowin, Beijing, China) as an internal control. Goat antibodies against mouse or rabbit IgG-HPR (1:8000, Cowin) were used as secondary antibodies. The blots were visualized using enhanced chemiluminescence (Cowin).

### 4.7. Statistical Analysis

Statistical analyses were conducted using a one-way ANOVA for each triplicate or quadruplicate sample set of data using SPSS version 17.0 (SPSS, SPSS Inc., Chicago, IL, USA). Values for all parameters are expressed as the mean ± SEM. A *p* value <0.05 was considered statistically significant.

## 5. Conclusions

In summary, the present work examined the hypothesis that STC1 inhibits Ca^2+^ transcellular transport in intestinal absorptive epithelia by blocking the influx of Ca^2+^ into the cells through down-regulation of *TRPV6* and *TRPV5*. Our findings imply that the mechanisms involved in STC-induced inhibition of Ca^2+^ transport processes may be similar in both fish and mammals. Indeed, our study is the first to show the direct effects of STC1 on mammalian intestinal epithelial calcium-transport proteins, and we hope that these findings enhance the recognition of the roles played by STC1 in mammals. Additionally, the possibility that STC1 influences cytosolic diffusion processes and the molecular mechanisms including calbindin-D_28K_ and -D_9K_ should not be ignored because they are also tightly controlled by various calciotropic hormones [2]. Future research should aim to resolve these questions.

## Figures and Tables

**Table 1:** Real-time primer sequences utilized in this study.

Gene	Genebank Accession No.	Primer (5′–3′)	Product Length (bp)	Annealing Temperature (°C)
*TRPV6*	NM_018646	GGACAACACCCTCTTACAGCA(sense)	224	60
		CCAGCACCATGAAGGCATA(anti-sense)		
*TRPV5*	NM_019841.4	TCTTAGGCAACTTCTACTGGACTG(sense)	223	60
		ACGCACCAGGTTCACATTCT(anti-sense)		
*PMCA1*	NM_001001323	CAGCAGGAGAACCAGAACCA(sense)	159	60
		CAGTGACCATCCGCACAGTAA(anti-sense)		
*NCX1*	XM_005264514.1	TGTGCATCTCAGCAATGTCA(sense)	230	60
		TTCCTCGAGCTCCAGATGTT(anti-sense)		
*VDR*	NM_000376	GTGGACATCGGCATGATGAAG(sense)	181	60
		GGTCGTAGGTCTTATGGTGGG(anti-sense)		
*GAPDH*	NM_002046	TGCACCACCAACTGCTTAGC(sense)	87	60
		GGCATGGACTGTGGTCATGAG(anti-sense)		
